# Use of Meat-Bone Paste to Develop Calcium-Enriched Liver Pâté

**DOI:** 10.3390/foods10092042

**Published:** 2021-08-30

**Authors:** Zhanibek Yessimbekov, Aitbek Kakimov, Nicola Caporaso, Anuarbek Suychinov, Baktybala Kabdylzhar, Mohammad Ali Shariati, Assemgul Baikadamova, Rubén Domínguez, José M. Lorenzo

**Affiliations:** 1 Department of Technological Equipment and Mechanical Engineering, Shakarim State University of Semey, Semey 071412, Kazakhstan; bibi.53@mail.ru (A.K.); baktybala.20@mail.ru (B.K.); asemgul93@yandex.ru (A.B.); 2 Department of Food Sciences, School of Biosciences, The University of Nottingham, Nottingham LE12 5RD, UK; nicola.caporaso3@unina.it; 3 Department of Agricultural and Food Sciences, University of Naples “Federico II”, 80055 Naples, Italy; 4 Kazakh Research Institute of Processing and Food Industry (Semey Branch), Semey 071410, Kazakhstan; asuychinov@gmail.com; 5 Department of Technology of Food Production, K.G. Razumovsky Moscow State University of Technologies and Management (The First Cossack University), 109004 Moscow, Russia; shariatymohammadali@gmail.com; 6 Centro Tecnológico de la Carne de Galicia, Avda. Galicia n° 4, Parque Tecnológico de Galicia, San Cibrao das Viñas, 32900 Ourense, Spain; rubendominguez@ceteca.net; 7 Área de Tecnología de los Alimentos, Facultad de Ciencias de Ourense, Universidad de Vigo, 32004 Ourense, Spain

**Keywords:** healthy meat products, mineral enrichment, calcium, functional meat products, meat by-products

## Abstract

The production technology of meat-bone paste and its effect on chemical, mineral and amino acid compositions of liver pâté were studied. The liver was replaced by meat-bone paste in the concentration of 5, 10, 15, 20, and 25% for the production of experimental samples. The compositional analysis of pâté manufactured with meat-bone paste showed that the reformulation did not influence the content of moisture (~56%), fat (~28%), or protein (~11%) while producing a significant increase of ash and a decrease of carbohydrates in comparison with control pâtés. The higher amounts of minerals of bone-meat paste, including calcium (3080 mg/100 g), magnesium (2120 mg/100 g), phosphorous (2564 mg/100 g), and iron (7.30 mg/100 g), explained the higher amount of both ash and these minerals in the reformulated samples compared to the control samples. The total caloric value (~300 kcal/100 g) was also unaffected by the addition of bone-meat paste. The content of both essential and non-essential amino acids decreased with the inclusion of meat-bone paste, although this decrease was lower in essential (6280 mg/100 g in control vs. 5756 mg/100 g in samples with 25% of meat-bone paste) than in non-essential amino acids (6080 mg/100 g in control vs. 3590 mg/100 g in samples with 25% of meat-bone paste). This fact is due to several essential amino acids not showing differences between control and reformulated samples, while in non-essential amino acids, these differences were greater. The results of this study showed that meat-bone paste addition is a good strategy to produce liver pâté enriched in minerals and with minimum influence on the content of the other important nutrients. Therefore, these results can be used for the design of new liver pâté with an increased nutritional significance by using meat industry by-products. According to the balance of minerals, the use of 15% of meat-bone paste to reformulate liver pâté is the best strategy used in the present research. However, additional studies on the stability (during storage), shelf-life, and sensory acceptability of these reformulated pâtés should be carried out.

## 1. Introduction

The composition of liver pâté can have a substantial impact on its nutritional characteristics, with a wide variety of liver pâté recipes reported in the literature and sold on the market worldwide. Such recipes can include beef liver, beef brain, and other beef components [1]; pork fat and other pig-derived products [2,3,4]; poultry fats and poultry-derived products [5]; and others [6]. Each of these components has unique chemical properties, flavors, and associated risks. In general, flavors derived from pork and beef are known to be more intense compared to poultry-derived analogues [7]. The food industry that manufactures liver pâté must consider all of these factors in designing a recipe, with the goal of obtaining a liver pâté that is safe, stable, rich in flavor, and appealing to consumers [8,9].

Of the aforementioned wide variety of ingredients that can be potentially used in liver pâté, meat-bone paste is of great interest [10]. Meat-bone paste has been shown to be rich in calcium and phosphorus [11] and as a result, has been studied as a possible dietary supplement to combat the ageing process [12]. The processing of meat-bones into such paste can affect its texture and the properties of the food to which is added, but amounts above 15% are known to have deleterious effects [13]. 

Among animal by-products, bones have almost no use in meat technology. This is because according to current legislation, bones are considered not suitable for human consumption and are treated as animal by-products [14]. Most small and medium meat companies dispose of them, whereas big companies produce bone adhesive, gelatin, bone flour, etc. However, bone is a rich source of mineral elements (calcium, phosphorous, magnesium, iron, etc.), protein (collagen type), and fat [15]. Therefore, although the characteristics of this product present promising advantages and opportunities for the reformulation of meat products, in addition to in-depth studies on its use, a change in legislation is also needed to include meat-bone paste as an ingredient for human consumption. This will undoubtedly require specific handling of the bones at the slaughterhouse level to ensure their safety. The improvement of bone processing technology and the creation of a new type of equipment allow using the bones of slaughtered animals for food purposes without losing nutrients. Mainly, in large meat companies, bone is used for the production of meat and bone meal for animal feeding. Meat and bone meal is a source of proteins as it contains essential amino acids which are vital for animal growth. Among the essential amino acids, the meat and bone meal contains large amounts of lysine, valine, isoleucine, and leucine [16].

Meat and meat products are widely consumed, and among them, pâtés are highly appreciated by consumers [6,17]. However, although meat contains high amounts of important nutrients (proteins, iron, vitamins, etc.), traditional meat products, such as pâté, do not provide the human body with a sufficient number of mineral elements, especially in relation to calcium. This is due to meat being characterized by typical low calcium contents (10–12 mg/100 g) and an unbalanced calcium to phosphorous ratio (1:15–20), as opposed to the recommended 1:1 ratio, leading to malassimilation of these elements [18]. With this in mind, the reformulation of pâtés using meat-bone paste as a raw material could allow the mineral supplementation and produce an enriched meat product. Some studies and patents were proposed for the isolation and hydrolysis of proteins from meat-bone paste [19,20]. However, these methods of bone processing are based on heating and acid treatment, which leads to some nutrient loss. To overcome these limitations, particular emphasis should be placed on the ultrafine grinding process of bones for food purposes. Ultrafine grinding of bone allows producing meat-bone paste that can be used in the formulation of sausage, pâtés, ham, meat semi-products, etc. Additionally, this meat-bone paste enriches meat products with essential mineral and protein substances. Ultrafine bone grinding processes begin by crushing the bone to 1–3 mm particles, followed by ultrafine grinding to yield 50–100 µm particles. This processing can be used to make paste-like products, such as pâté, that have a soft texture and are fully digestible by humans. Moreover, since the meat-bone grinding process does not involve thermal treatments, the vitamin and mineral content is preserved. The use of grinding and ultrafine grinding technology for obtaining meat-bone pastes was previously reported by several authors and companies [21,22,23]. Additionally, the use of meat-bone paste for the development of functional meat products was also studied [24,25].

Thus, the aim of the current study was to apply technology for manufacturing liver pâté made with meat-bone paste in order to investigate its impact on the chemical composition of the final product. The liver pâté was made with a meat-bone paste at different levels (from 5 to 25% of the total ingredients), and other ingredients included beef liver, beef brain, pork back fat, sautéed onion, salt, white sugar, and other spices. A detailed analysis of the chemical composition, minerals, and amino acid content of the liver pâté was carried out.

## 2. Materials and Methods

### 2.1. Meat-Bone Paste Preparation

A patent was granted by the Republic of Kazakhstan #2202 on 15 June 2017 for the method developed by Kakimov et al. [15]. Bone grinding processing by this procedure allows obtaining a meat-bone paste which is free of hard bone particles; thus, it results in a product that is smooth and soft to the tongue of the consumer. 

Beef tissue and bones (rib and vertebrae) were donated by the Tyumenbayev Meat Company in Semey city, Republic of Kazakhstan. Meat and rib bones from cattle were obtained after carcass boning and were packed in clean polyethylene bags that were rapidly transported to the laboratory and stored at −18 °C for 2 h. The total weight of bone and meat was 50 kg (25 kg each).

Bones with meat tissue were washed with cold water and then crushed into 50–70 mm long fragments. Cutting bones into small pieces was done manually with an axe. The bone fragments were stored at −18 °C to −20 °C before loading into the hopper of a crushing machine equipped with an 8 mm diameter meat grinder plate. The bone was ground and crushed again using a 3 mm meat grinder plate; water was then added to a 1:1 ratio (*w*/*w*). The mixture was frozen at −3 °C to −5 °C for 1 h and then ground using a micro-milling machine having rotational knives spaced at 0.50 mm. The resulting meat-bone paste (MBP) was used to prepare pâté meat batters. 

### 2.2. Liver Pâté Preparation

**Figure 1:**
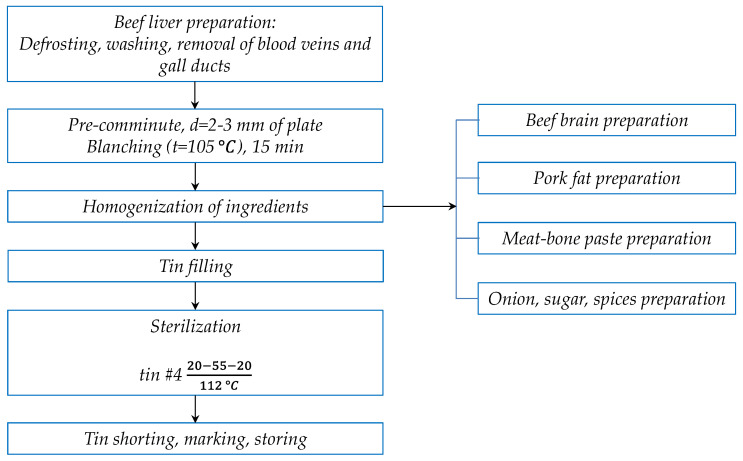
Flow sheet of liver pâté preparation.

As a control, we used meat pâté with a formulation approved and reported in National Standard GOST 12319-77 [26]. In other samples, the beef liver was replaced by meat-bone paste in amounts ranging from 5 to 25% (Table 1). The manufacturing steps and conditions are summarized in Figure 1. All ingredients were inspected for safety in accordance with existing specifications and standards. Then, beef liver was carefully trimmed, removing the membrane, bile ducts, and other inclusions. After trimming, the beef liver was soaked in running water for 2 h for removing blood clots. At that point, the liver was cut into pieces and blanched in hot water (water/liver ratio 3:1) for 25 min. After blanching, the liver was rinsed in cold water and chopped in a meat grinder with 2–3 mm hole diameter of plate. Beef brains were used for pâté elaboration after veterinary and sanitary inspection, in accordance with regulations [27]. Beef brains were blanched for 10–15 min and cooled to 12 °C. After cooling, the beef brains were chopped on the meat grinder with 2–3 mm hole diameter of plate. Then, beef liver, beef brains, meat-bone paste, pork fat, and spices were weighed according to the recipe (Table 1). The ingredients of the pâté were mixed in a cutter L5-FKM (Russia) for 5–7 min. 

The pâté mass was filled by dosing machines into metal cans (height 70.0 mm; diameter 72.8 mm; volume 260 mL) and sealed hermetically on the capping machine IPKS-127UZ (Moscow, Russia). The net weight of cans was 250 g. Capped cans after washing were loaded into autoclave baskets and sent for sterilization in autoclave IPKS-128-500 (Moscow, Russia). The time from closing the cans to the beginning of sterilization should not exceed 30 min. Sterilization was carried out using the following program: initial 20 min to reach the treatment temperature in the autoclave (112 °C; heating step), then maintaining cans for 65 min at 112 °C and 0.08 MPa (treatment conditions), and finally another 20 min to lower the temperature (cooling step). 

All manufacturing processes were replicated twice (with the same ingredients and processing conditions), and five cans of each batch and replicate were analyzed for the chemical, mineral, and amino acid composition. 

### 2.3. Determination of Chemical Composition

The determination of the chemical composition (moisture, fat, ash, and protein) was based on the methods previously described [23]. Briefly, to determine moisture, the samples were dried in a drying oven, and moisture was calculated according to the standards [28,29]. After moisture determination, each dried sample was used for fat determination according to the standard GOST 23042-86 [30]. To measure the ash content, the samples were calcined in a muffle furnace (500 °C–600 °C). Finally, the protein content was analyzed according to the standard GOST 25011-81 [31].

The method used for carbohydrate determination is based on extraction of sugars from the analyzed sample with distilled water followed by HPLC analysis to identify the composition and determine the carbohydrate mass fraction. Briefly, 10 g of pâté samples was mixed with 100 mL of distilled water, and the obtained mixture was mixed with a magnetic stirrer at room temperature for 1 h. The obtained extract was filtered through a paper filter and then through a 0.45 µm membrane filter. The prepared extract was then analyzed by HPLC.

The carbohydrates were quantified using an HPLC (Shimadzu LC-20 Prominence liquid chromatography system; Shimadzu, Kyoto, Japan) equipped with a refractive index detector (RID). For carbohydrate determination, 20 µL of each sample was injected, and carbohydrates were separated using column with aminopropyl stationary phase for carbohydrate separation (Zorbax Carbohydrate 250 × 4.6 mm, 5 μm, Agilent, Santa Clara, CA, USA), using acetonitrile:water in proportion 82%:18% (*v*/*v*) as eluent at 0.5 mL/min in isocratic mode. The identification of carbohydrates was carried out by comparison individual peaks with the retention times of pure standards. The total analysis time was 20 min. The concentration of each carbohydrate was calculated with an external calibration, and the results of total carbohydrate content was expressed as g/100 g of pâté. 

### 2.4. Caloric Value Calculation

Caloric value was calculated according to Equation (1):

(1) CV=4 (P+C)+9F

where *CV*—caloric value, kCal; *P*—protein content, g; *F*—fat content, g; *C*—carbohydrate content, g; 4—caloric index for protein and carbohydrate; and 9—caloric index for fat.

### 2.5. Mineral Composition Determination 

One to two grams of the sample was placed in a high-pressure Teflon container. Each sample was combusted at 400 °C for 4 h and then to 600 °C for 2 h using a muffle furnace. A representative 1 g (dry weight) of ashes was digested by adding 3 mL HNO_3_ and 2 mL of HF. This was placed in a microwave at 200 °C for 20 min (Berghof Speed Wave microwave system, Bremen, Germany). After microwave digestion, the samples were diluted with 1% HNO_3_ in a 10 mL vessel.

The content of elements in muscle samples was determined with an inductively coupled plasma mass spectrometric method (ICP-MS, Varian-820 MS, Varian Company, Canberra, Australia). The method was validated with certified reference materials. Calibration standards Var-TS-MS, IV-ICPMS-71A (Inorganic Ventures Company, Christiansburg, VA, USA) were used for calibrating the mass spectrometer. The sensitivity of the mass spectrometer was tuned using a diluted calibration solution Var-TS-MS with a concentration of Ba, Be, Ce, Co, B, Pb, Mg, Tl, and Th of 10 µg/L. Three calibration solutions were used for the detector calibration. They were IV-ICPMS-71A of Cd, Pb, Cu, and Zn elements diluted to 10, 50, and 100 µg/L. Discrepancies between the certified values and concentrations quantified were below 10%. The operating parameters of the inductively coupled plasma mass spectrometer Varian ICP 820–MS were as follows: plasma flow 17.5 L/min; auxiliary flow 1.7 L/min; sheath gas 0.2 L/min; nebulizer flow 1.0 L/min; sampling depth 6.5 mm; RF power 1.4 kW; pump rate 5.0 rpm; and stabilization delay 10.0 s. All analyses were performed in triplicates, and the results were expressed as mg/100 g sample.

### 2.6. Amino Acid Composition Determination

Determination of the amino acid composition of meat samples was performed based on the phenylisothiocyanate (PITC) procedure described by Rudenko and Kartsova [32]. For determination of amino acid composition in meat samples, 100 mg of homogenized sample was mixed with 10 mL of 6M hydrochloric acid in vials under nitrogen atmosphere. Then, vials were closed and turned to the thermostat with temperature 110 °C for at least 17 h. Then, hydrolyzed solutions were cooled to room temperature and filtered. Measurements of 0.5 mL of the filtered solutions were dried in the airflow at 65 °C. To the dried aliquots, 100 µL of 0.15 M solution of sodium hydroxide was added and stirred for 10 min. After this, 350 µL of phenylisothyocyanate (PITC) solution in isopropyl alcohol and 50 µL of water were added. Solutions were stirred and dried at 65 °C in the airflow and water bath. Dry residues of solutions were dissolved in 1 mL of water and filtered through 0.45 μm filters. For identification (by retention times) and quantification (based on external standard calibration), the commercial standards of the amino acids (Asp, Asn, Glu, Gln, o-Pro, Ser, Gli, Gis, Arg, Tre, Ala, Pro, Tir, Val, Lys, Ile, Ley, Phe, Met, Cys) were derivatized using PITC procedure as described below. The amino acid derivatives were analyzed using a Shimadzu Prominence LC-20 liquid chromatograph equipped with 250 × 4 mm Supelco C18 (5 μm) column and UV detector at wavelength 254 nm [32]. Gradient mode (Table 2) at 1.2 mL/min eluent flow and 40 °C of column temperature was used. As mobile phase, 6 mM solution of sodium acetate with pH 5.5 (eluent A), 1% (*v*/*v*) solution of isopropyl alcohol in acetonitrile (eluent B), and 6 mM solution of sodium acetate with pH 4.05 (eluent C) were used.

### 2.7. Microstructure Analysis

Microstructure of meat-bone paste was observed by low vacuum scanning electron microscope JSM-6390LV JEOL (Tokyo, Japan), following the procedure described by Rao et al. [33].

### 2.8. Statistical Analysis

Statistical analysis was performed using Statistica 12.0 (STATISTICA, 2014; StatSoft Inc., Tulsa, OK, USA). After checking normal distribution and variance homogeneity (Shapiro–Wilk), the differences between liver pâté samples were evaluated using one-way ANOVA. The Tukey HSD test was used for means comparisons. Differences were considered to be statistically significant at *p* ≤ 0.05. Data are presented as mean values ± standard deviation (SD). 

## 3. Results and Discussion

### 3.1. Mineral Composition of Meat-Bone Paste

In order to determine the chemical characteristics of the meat-bone paste used for the reformulation of liver pâté, an initial characterization of it was carried out. The chemical composition of meat-bone paste was characterized by high ash content (11.4%), while the protein, fat, and moisture contents were 13.6%, 5.22%, and 69.7%, respectively (Table 3). The calculated caloric value of the meat-bone paste was 102 kcal. In another study, the authors also found high amounts of ashes in meat-bone paste [34]. However, these authors reported higher amounts of ash (29%), protein (24%), and fat (12%) and lower values of moisture (34%) than those observed in our study, which could be related to differences in the initial material used for the production of meat-bone paste. 

Bones are tough and hard tissue as a result of the combination of mineral elements with organic structure. Bone tissue is a rich source of mineral salts. It contains 98% of all inorganic salts presented in the human body, and among them, 99% of calcium, 87% of phosphorous, 58% of magnesium, and 46% of sodium [16]. Therefore, as expected, the mineral composition of meat-bone paste used in this study was dominated by calcium (3080 mg/100 g), phosphorous (2564 mg/100 g), magnesium (2120 mg/100 g), sodium (390 mg/100 g), potassium (115 mg/100 g), and iron (7.30 mg/100 g). Trace elements of copper (1.33 mg/100 g), manganese (0.10 mg/100 g), and zinc (2.15 mg/100 g) were also identified (Table 3). Thus, the analysis of meat-bone paste demonstrated high amounts of important minerals for human nutrition. The mineral composition found in the meat-bone paste used in the present study agrees with that reported by other researchers in a protein compound batter made with meat-bone paste and other meat by-products [35]. In fact, in this study, the authors reported that the most important minerals in this protein compound are higher amounts of calcium, phosphorous, magnesium, sodium, and potassium. Similar to our findings, other researchers also reported high amounts of calcium (57.7%), phosphorous (25.4%), and sodium (3.80%), while the content of magnesium (1.86%) and potassium (1.12%) was lower [36].

### 3.2. Microstructure Analysis of Meat-Bone Paste 

**Figure 2:**
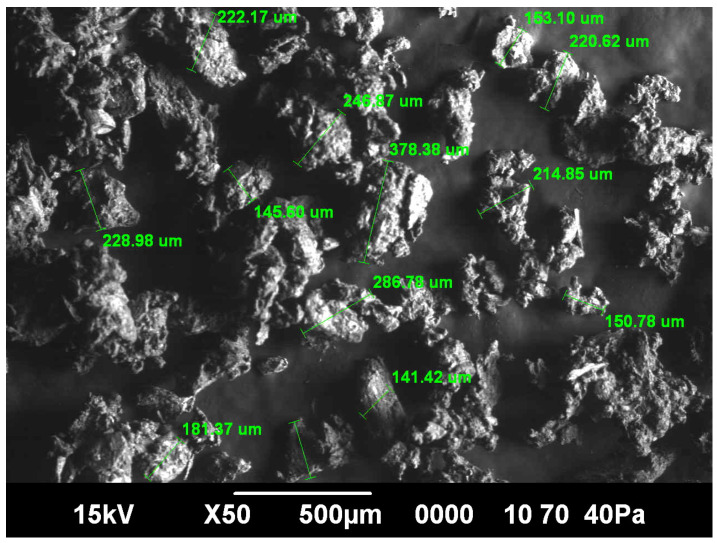
Bone particle sizes of meat-bone paste.

As can be seen in the image of bone particles, magnified 50 times where the bone particle sizes were measured (Figure 2), particle sizes exceeding 0.40 mm (400 microns) were not detected. 

On the basis of the sieve analysis of the meat-bone paste after grinding on a colloid machine with a gap between the grinding wheels of 0.10 mm, it was found that the mass fraction of bone particles ranging in size from 0.10 mm to 0.25 mm is more than 95%. Bone particles that were beyond 0.25 mm were less than 5% and, as mentioned, particles of 0.40 mm (or higher) were not detected. Similar findings were obtained in a previous study, in which the meat-bone particle size after grinding on the colloid mincing machine was from 0.20 to 1.5 mm, while after grinding on the superfine machine, the particle size was reduced to less than 0.10 mm [34]. A more recent study concluded that after grinding in the masscolloider with a gap of 0.25 mm, the bone particle size ranged between 0.14 mm and 0.37 mm, while after using a masscolloider with a gap of 0.10 mm, the bone size decreased and ranged between 0.045 mm and 0.19 mm [13]. These results agree with our findings, and they demonstrate that the process and conditions for obtaining the meat-bone paste are good and that this allows obtaining a meat-bone paste with a smooth texture, which is not perceptible by consumers, and which is digestible by humans. Therefore, the meat-bone paste obtained in this research can be used for the production or reformulation of meat products: in our case, liver pâté.

### 3.3. Liver Pâte Chemical and Mineral Composition

The analysis of chemical composition showed similar values of moisture (~56%), protein (~11%), and fat (~27%) compared to those reported by other authors concerning liver pâté [3,6,17] and slightly different (lower protein and higher fat contents) in comparison with meat pâtés [4]. In the case of pâté, as in other meat products, the final composition of the product depends on the composition, type, and proportions of the ingredients used during processing. Therefore, the differences found between the studies could be attributed to the ingredient composition and the specific formulation used for pâté manufacturing in each study. 

In the present study, the addition of meat-bone paste significantly affected the content of ash and carbohydrate, while it did not influence any other parameters (Table 4). Although the protein values were not significantly influenced (*p* > 0.05) by the reformulation, a slight and progressive decrease in its content could be observed as the meat-bone paste proportion increased. The lower amount of protein in meat-bone paste (13.6%) in comparison with liver protein content (18.9%) [37] could be the explanation for this behavior. Fat and moisture contents were constant in all pâté samples. The similar fat content between meat-bone paste and beef liver (5.22% in meat-bone paste vs. 3.80% in the beef liver [37]) could explain the lack of significant differences in this parameter when liver pâté is reformulated with meat-bone paste. In contrast, the ash content increase from 0.92% (control) to 3.42% (BMP-25) could be explained by the high amount of minerals in the meat-bone paste. As discussed in the previous section, the meat-bone paste used in the present study presented high ash content (11.4%) in comparison with 1.80% of ash found in beef liver [37], which exerted a clear influence on the evolution of these parameters when the pâté was reformulated. Finally, the carbohydrate content was also affected by the inclusion of meat-bone paste as a liver replacer. In this case, a progressive decrease of carbohydrates was observed as the proportion of meat-bone paste used in the pâté formulation increased. 

Similar results were obtained in previous studies, in which beef meat was partially (from 10% to 40%) replaced by meat-bone paste in meat batters [15]. In this case, the authors observed that moisture and protein contents were not affected by the reformulation. However, according to our results, these authors also observed a progressive (not significant) decrease in protein content when increasing the meat-bone paste proportion. Due to meat-bone paste presenting a high content of ash, in the previous study, the authors also reported a dramatic increase in the ash content of meat batter when meat-bone paste was used as a meat replacement.

The calculated caloric value of liver pâtés with meat-bone paste varies from 299 kcal/100 g (MBP-15) to 306 kcal/ 100 g (MBP-5) and is not so different. However, the caloric value of the control pâté was slightly higher 307 kcal/100 g. It is important to highlight that the differences between caloric values were not significant, and these values were similar among all treatments. This fact is related to the very similar caloric value of both beef liver and meat-bone paste (106 vs. 110 kcal/100 g in meat-bone paste and liver, respectively). In contrast to our finding, in another study, the authors reported a significant reduction of energy value when the meat-bone paste replaced beef meat in meat batters [15]. In this case, this significant reduction was directly related to the reduction of fat content in the reformulated samples, while in our study, as aforementioned, the fat content was constant in all samples, which explains the different behaviors in these studies.

The mineral profile of liver pâtés with meat-bone paste is shown in Table 5. As expected, the replacement of liver with meat-bone paste has significantly increased calcium, magnesium, phosphorous, and sodium content. Additionally, the iron content was significantly higher in MBP-20 and MBP-25 samples in comparison with the other batches. These results are directly related to the mineral composition of meat-bone paste, which, in accordance with previously published articles, presented very high amounts of these minerals. Therefore, as mentioned above, our findings reflect the change in both ash content and mineral composition with the reformulation.

On the contrary, the low content of potassium, copper, manganese, and zinc in meat-bone paste determines that the content of these minerals decrease as the meat-bone paste replaced beef liver in the formulation of the reformulated pâtés. 

As discussed, mineral profile analysis showed that samples MBP-20 and MBP-25 had the highest concentrations of calcium, phosphorous, and sodium compared to other samples. Thus, consumption of 100 g of liver pâté with 20 and 25% of meat-bone paste provides more than 50% of the recommended dietary allowance (RDA) of calcium and phosphorous to the human body (Table 6). However, excessive amounts of these elements can lead to serious diseases [38]. The amount of calcium increased from 11.4 mg/100 g in the control sample to 780 mg/100 g in MBP-25 (Table 5). Calcium is the main component of bone tissue and dentin and is vital for the healthy performance of nervous and cardiovascular systems and for the metabolism to help regulate the acid–base balance [39]. Calcium deficiency in the human body causes diseases such as osteoporosis, chronic illness of skeleton, and bone tissue. Moreover, hypertension symptoms are also related to calcium deficiency. 

The magnesium level increased three times from 12.6 mg/100 g in the control sample to 37.7 mg/100 g in liver pâté with 25% MBP. Magnesium is another essential element in the human body. Magnesium ions participate in more than 350 different biochemical processes, including the function of adenosine triphosphate, protein, carbohydrate, and lipid metabolisms and stabilizing enzymes [40]. Magnesium deficiency can result in the activation of inflammatory conditions and lead to oxidative damage of tissues, increased risk of hypertension, and heart diseases [41]. However, magnesium abundance impairs calcium bioavailability. 

The sodium content in the reformulated liver pâtés was found to be significantly higher than that of the control sample. Sodium plays a key role in water and electrolyte balance in the human body. However, excess sodium intake was related to multiple illnesses [42,43,44], such as slowing down body growth, raising blood pressure, and leading to heart and kidney dysfunctions. 

Phosphorus compounds are presented in every cell of the human body and participate in the functioning of live activities. Therefore, the liver pâté reformulation produces an enrichment in this mineral. It provides energy for muscle actions, nervous impulses, and organic matter biosynthesis. Phosphorus plays an important role in calcium metabolism, brain functioning, muscle, and bone strength and is a part of DNA, RNA, and several ferments [45]. However, an excess amount of phosphorous can cause calcium loss and the deposit of mineral salts in muscles [46].

Iron is an integral component of the blood-forming process. It helps to produce red and white blood cells. Anemia is the most common disease of iron deficiency [47]. Excess iron has a bad effect on liver function [48].

However, among the minerals that reduce their content with the reformulation (K, Cu, Mn, and Zn), the highest reduction (up to 40%) was observed for copper, while manganese, potassium, and zinc levels were reduced by 30%, 23%, and 22%, respectively. Potassium level was lower when increasing meat-bone paste in liver pâtés. Potassium functions as an intracellular ion, regulating the water, acid, and electrolytic balance and helps to lower blood pressure. Its deficiency has been linked to serious illnesses, such as cardiac failure, depressive syndrome, nightmares, kidney failure, etc. [49]. However, an excess level of potassium in the human body can lead to heart dysfunction, weakness, or adynamia [50].

Copper participates in iron metabolism and cell respiration and stimulates protein and carbohydrate digestibility. Copper is part of many vitamins, enzymes, and hormones [51].

Manganese is essential in forming bone and connective tissue. It is a part of many enzymes that participate in amino acid, carbohydrate, and catecholamine metabolism, stimulating cholesterol biosynthesis [52]. Lack of manganese in the human body causes different pathologic processes: a decrease of antibody response rate; neuropsychic disease; diabetes; brittleness of the bones; etc. [53].

Zinc is a vital microelement for the normal functioning of the human body. More than 300 enzymes contain zinc. This element participates in the synthesis and degradation of proteins, carbohydrates, fats, and nucleic acids [54]. Zinc deficiency could cause anemia, secondary immunodeficiency, and liver cirrhosis [55]. An excessive amount of zinc in the human body predisposes one to acute intoxication. 

On the other hand, similar studies reported the calcium fortification of meat products. The mineral source supplements used are different types of bones (animal, poultry, fish) and mineral supplements. For example, Chinese scientists from Chengdu University and Sichuan Tianxian Food Co., Ltd. (Yan’an, China), patented a method of meat food products enriched with minerals, in particular calcium and phosphorous [56]. The source of mineral elements was the bones of animals, fish, and poultry, fish and poultry bones being treated with alkaline and acid solutions and heated. After the bones were finely crushed, an enzymatic treatment was performed to obtain a paste-like mass. This paste-like mass was used in formulations of meat products which allows for enriching with biologically active calcium and phosphorus [56]. 

Boyle et al. [57] developed low-fat sausages enriched with calcium carbonate and calcium citrate malate complex. In this case, the content of calcium in low-fat sausages enriched with calcium carbonate was approximately 550 mg/100 g, and calcium citrate malate complex was 510 mg/100 g. The current data obtained in our research for liver pâté with 15% of meat-bone paste were in agreement with the previous data [57]. 

Cáceres et al. [58] used calcium lactate, calcium gluconate, and calcium citrate in the formulation of cooked sausages. The concentration of calcium in sausages was up to 350 mg/100 g. Fishbone powder processing technology was also reported [59]. Ustinova et al. [60] patented the production method of emulsified meat products enriched with minerals. The increase in calcium content (227 mg/100 g) was attributed to the addition of bone powder into the formulation of the meat products. Daengprok et al. [61] used eggshell calcium lactate powder in a recipe of Thai-style fermented pork sausage and observed that calcium supplementation should be limited to 150 mg/100 g (equivalent to 18.8% of the RDA for adults) [61]. Another study developed low-fat chicken patties fortified with 1.75% of calcium lactate and determined 213 mg/100 g of calcium concentration, achieving 20% of RDA of calcium. 

Therefore, according to our results of mineral composition, the optimal level of meat-bone paste addition to liver pâté formulation is 15%. This amount provides the most balanced ratio of calcium to phosphorous (1:1.2) and the beneficial effect of better assimilation of calcium in the human body [62]. 

### 3.4. Amino Acid Composition 

Adding meat-bone paste had a dramatic effect on the amino acid composition of liver pâté (Table 7). 

Replacing 20 or 25% of the liver with the same amount of meat-bone paste significantly increased the content of histidine (from 409 mg/100 g in the control sample up to 454 mg/100 g in the MBP-25 sample). The same trend was obtained for lysine and isoleucine, but in these cases, the differences were not significant. 

The content of the other amino acids decreased when increasing amounts of meat-bone paste. For the most part, all non-essential amino acids were reduced on average by 40%, except for oxyproline (reduced by more than 45%). Among the essential amino acids, the average reduction was 5%, except for threonine (17%) and tryptophane (41%).

The reduction of total non-essential amino acids was 40.9% (6080 vs. 3590 mg/100 g in control and MBP-25, respectively), while the total essential amino acids only decreased by 8.30% (6280 vs. 5756 mg/100 g in control and MBP-25, respectively). This aspect is very important from a nutritional point of view since essential amino acids participate in the synthesis of proteins and have a direct effect on weight gain. Moreover, each one performs its own specific functions. Lysine and tryptophan are required for growth; lysine with histidine participates in blood formation [63]. Methionine has a great effect on fat and phosphatide metabolism, which plays a role in nervous functions. Cystine has a strong antioxidant activity which increases with the presence of vitamin C and selenium; goat milk has a high content of both [64]. Leucine, isoleucine, and valine are the basic amino acids for protein metabolism. These amino acids are the main source of energy for the muscles, reduce the rate of protein breakdown, and support muscle mass and protein synthesis during physical stress [65].

In observing the reduction in total amino acid content (from 12,360 mg/100 g in control to 9345 mg/100 g in MBP-25), as well as the reduction in protein content discussed above, a decrease in amino acid content was expected. In fact, our results agree with those reported in a previous study, in which the authors found a significant decrease of total, essential, and non-essential amino acids in meat batters reformulated with 10–40% of meat bone paste [15]. However, in the present study, the particular amino acid composition of meat-bone paste [16], rich in valine (8.10% of protein), isoleucine (7.40 % of protein), leucine (12.5% of protein), and lysine (13.6% of protein), demonstrates that the reformulation did not influence the content of these important essential amino acids. In comparison, according to a recent paper [37], the content of these amino acids in beef liver (3.51, 5.67, 7.99, and 7.37% of protein for valine, isoleucine, leucine, and lysine, respectively) is lower than the values reported for meat-bone paste, which explains our findings in this research. 

## 4. Conclusions 

The liver pâté developed by using meat-bone paste yielded a high-quality food product enriched with minerals, such as calcium, magnesium, and iron, satisfying up to 50% of the normal RDA. This innovative type of liver pâté expands the range of canned foods on the market and increases the cost efficiency by using lower-cost ingredients obtained from using meat processing industry by-products. Moreover, the consumption of liver pâté with meat and bone paste might be beneficial for the issue of mineral deficiency in the human body and osteoporosis disease prevention. This study showed that partial replacement of liver with meat-bone paste significantly increases the calcium, magnesium, sodium, and phosphorous content in liver pâtés. According to the mineral balance, the optimal amount of meat-bone paste in pâté formulation should be up to 15%. In this case, the consumption of 100 g of pâté provides 50% of the RDA of calcium and phosphorous to the human body.

In general, the changes in chemical compositions did not affect the main macronutrients (fat and protein) of the product, while the amino acid composition changed with the reformulation. In this regard, a dramatic decrease of the non-essential amino acids was found, but the change in essential amino acids content was limited, thus confirming that the use of meat-bone paste as an ingredient is good for producing healthy pâtés. Therefore, partial replacement of beef liver meat-bone paste reduces production costs and offers the possibility of reusing nutritive by-products of the meat industry, while enriching meat batters with minerals and essential amino acids.

## Figures and Tables

**Table 1:** Formulations of liver pâté with different amount of meat-bone paste (%).

Ingredients	Samples					
	Control	MBP-5	MBP-10	MBP-15	MBP-20	MBP-25
Meat-bone paste	0.00	5.00	10.0	15.0	20.0	25.0
Beef liver	55.0	50.0	45.0	40.0	35.0	30.0
Beef brain	10.0	10.0	10.0	10.0	10.0	10.0
Pork back-fat	30.0	30.0	30.0	30.0	30.0	30.0
Sauteed onion	3.10	3.10	3.10	3.10	3.10	3.10
Salt	1.30	1.30	1.30	1.30	1.30	1.30
White sugar	0.40	0.40	0.40	0.40	0.40	0.40
Spices *	0.20	0.20	0.20	0.20	0.20	0.20
Total	100	100	100	100	100	100

* Black pepper and allspice; nutmeg; cinnamon; cloves (in equal quantity).

**Table 2:** Gradient mode parameters of HPLC determination of amino acids.

Time (min)	Volume of Eluent (%)		
	Eluent A	Eluent B	Eluent C
0.01	96.0	4.00	0.00
10.0	37.0	11.0	52.0
13.0	88.5	11.5	0.00
21.0	80.0	20.0	0.00
22.0	58.0	22.0	20.0
24.0	0.00	24.0	76.0
32.0	0.00	33.5	66.5
32.01	20.0	80.0	0.00
35.3	20.0	80.0	0.00
35.31	97.0	3.00	0.00
41.3	97.0	3.00	0.00

**Table 3:** Chemical (g/100 g) and mineral composition (mg/100 g) of meat-bone paste.

Chemical Composition	
Moisture	69.7 ± 1.57
Protein	13.6 ± 0.32
Fat	5.22 ± 0.14
Ash	11.4 ± 0.36
Caloric value (Kcal/100 g)	102
Minerals	
Calcium	3080 ± 72.7
Potassium	115 ± 1.86
Magnesium	2120 ± 43.5
Sodium	390 ± 14.8
Phosphorous	2564 ± 50.4
Copper	1.33 ± 0.04
Iron	7.30 ± 0.14
Manganese	0.10 ± 0.002
Zinc	2.15 ± 0.08

**Table 4:** Chemical composition of liver pâtés with meat-bone paste.

Parameter ^1^	Control	Pâté Reformulated with Meat-Bone Paste					*p* Value
		MBP-5	MBP-10	MBP-15	MBP-20	MBP-25	
Moisture	56.9 ± 1.27	56.8 ± 1.59	56.7 ± 1.27	57.0 ± 0.93	56.5 ± 1.71	56.4 ± 1.58	>0.50
Protein	11.3 ± 0.46	11.1 ± 0.21	10.9 ± 0.21	10.7 ± 0.30	10.5 ± 0.26	10.2 ± 0.23	>0.25
Fat	27.7 ± 0.80	27.7 ± 0.71	27.8 ± 0.56	27.2 ± 0.98	28.0 ± 0.78	28.0 ± 0.73	>0.50
Ash	0.92 ± 0.02 ^f^	1.42 ± 0.04 ^e^	1.92 ± 0.04 ^d^	2.36 ± 0.07 ^c^	2.92 ± 0.08 ^b^	3.42 ± 0.08 ^a^	<0.05
Carbohydrates	3.25 ± 0.07 ^a^	2.98 ± 0.07 ^a^	2.72 ± 0.05 ^b^	2.68 ± 0.07 ^b^	2.19 ± 0.06 ^c^	1.92 ± 0.05 ^c^	<0.05
Caloric value	307	306	305	299	302	301	

^a–f^ means within the same row with different uppercase letters differing significantly among different samples of liver pâtés (*p* < 0.05); ^1^ caloric value results are expressed as kcal/100 g, while the chemical composition data were expressed as g/100 g.

**Table 5:** Mineral composition (expressed as mg/100 g) of liver pâtés with meat-bone paste.

Mineral	Control	Pâté Reformulated with Meat-Bone Paste					*p* Value
		MBP-5	MBP-10	MBP-15	MBP-20	MBP-25	
Calcium	11.36 ± 0.19 ^f^	165 ± 4.21 ^e^	319 ± 7.81 ^d^	500 ± 18.7 ^c^	626 ± 10.9 ^b^	780 ± 14.1 ^a^	<0.001
Potassium	211 ± 7.30 ^a^	201 ± 4.82 ^a^	191 ± 1.81 ^b^	181 ± 5.91 ^b^	171 ± 6.04 ^b,c^	161 ± 4.65 ^c^	<0.01
Magnesium	12.6 ± 0.27 ^e^	17.7 ± 0.39 ^d^	22.7 ± 0.64 ^d^	25.0 ± 0.76 ^c^	32.7 ± 0.73 ^b^	37.7 ± 0.74 ^a^	<0.001
Sodium	61.5 ± 2.08 ^f^	77.4 ± 1.52 ^e^	93.2 ± 1.76 ^d^	109 ± 3.17 ^c^	125 ± 2.50 ^b^	141 ± 4.39 ^a^	<0.001
Phosphorous	275 ± 7.37 ^f^	382 ± 12.6 ^e^	489 ± 8.56 ^d^	596 ± 10.8 ^c^	703 ± 11.7 ^b^	810 ± 18.4 ^a^	<0.001
Copper	6.99 ± 0.21 ^a^	6.42 ± 0.15 ^a^	5.86 ± 0.19 ^b^	5.29 ± 0.16 ^b^	4.72 ± 0.11 ^c^	4.16 ± 0.13 ^c^	<0.01
Iron	4.19 ±0.11 ^b^	4.20 ±0.10 ^b^	4.32 ±0.12 ^b^	4.59 ±0.09 ^b^	4.64 ±0.16 ^a^	4.86 ±0.12 ^a^	<0.01
Manganese	0.20 ± 0.01 ^a^	0.19 ± 0.01 ^a^	0.17 ± 0.01 ^a^	0.16 ± 0.01 ^b^	0.15 ± 0.01 ^b^	0.14 ± 0.01 ^b^	<0.01
Zinc	3.09 ± 0.08 ^a^	2.95 ± 0.08 ^a^	2.82 ± 0.09 ^a^	2.68 ± 0.05 ^b^	2.54 ± 0.07 ^b^	2.40 ± 0.06 ^b^	<0.01

^a–f^ means within the same row with different uppercase letters differing significantly among different samples of liver pâtés (*p* < 0.05).

**Table 6:** Recommended daily allowance of minerals, mg/day.

Mineral	Recommended ^1^	Maximum ^2^	Recommended for Children ^3^
Calcium	800–1200	2500	400–1200
Magnesium	300–400	-	55–400
Potassium	1500–2500	-	400–2500
Sodium	3000–5000	-	200–1300
Phosphorous	550–1400	-	300–1200
Iron	8–10 (for men) 15–20 (for women)	-	4–18
Zinc	11–15 (for men) 10–12 (for women)	25	3–12
Copper	0.9–2.3	5	0.5–1.0
Manganese	2–5	5	2

^1^ Recommended daily allowance (mg/day); ^2^ maximum acceptable concentration (mg/day); ^3^ recommended daily allowance for children (mg/day).

**Table 7:** Amino acid composition (expressed as mg/100 g) of liver pâté.

Amino Acid	Control	Pâté Reformulated with Meat-Bone Paste					*p* Value
		MBP-5	MBP-10	MBP-15	MBP-20	MBP-25	
*Non-essential*	*6080 ± 109*	*4995 ± 89.9*	*5083 ± 91.5*	*4586 ± 82.5*	*4088 ± 73.6*	*3590 ± 64.4*	
Alanine	718 ± 7.71 ^a^	583 ± 8.72 ^b^	602 ± 10.1 ^b^	544 ± 6.41 ^b^	485 ± 5.15 ^c^	427 ± 5.29 ^c^	<0.001
Aspartic acid	1177 ± 24.9 ^a^	967 ± 18.2 ^b^	984 ± 11.8 ^b^	888 ± 14.6 ^c^	792 ± 10.5 ^d^	695 ± 8.24 ^e^	<0.001
Glycine	702 ± 9.12 ^a^	583 ± 7.38 ^b^	586 ± 7.65 ^b^	528 ± 6.73 ^b^	469 ± 5.46 ^c^	411 ± 4.70 ^d^	<0.001
Glutamic acid	1585 ± 19.6 ^a^	1312 ± 16.8 ^b^	1324 ± 19.3 ^b^	1193 ± 12.6 ^c^	1063 ± 12.3 ^c^	932 ± 14.7 ^d^	<0.001
Proline	603 ± 11.2 ^a^	481 ± 7.30 ^b^	506 ± 7.15 ^b^	458 ± 6.51 ^b^	410 ± 4.36 ^c^	362 ± 4.62 ^d^	<0.001
Serine	555. ± 10.5 ^a^	454 ± 6.55 ^b^	465 ± 9.40 ^b^	419 ± 8.36 ^b^	374 ± 5.66 ^c^	329 ± 6.72 ^d^	<0.001
Tyrosine	483 ± 9.20 ^a^	405 ± 5.88 ^b^	402 ± 5.63 ^b^	362 ± 3.92 ^c^	322 ± 4.10 ^c^	281 ± 4.38 ^d^	<0.001
Cystine	232 ± 3.50 ^a^	188 ± 3.37 ^b^	194 ± 2.57 ^b^	175 ± 2.43 ^b^	157 ± 2.49 ^c^	138 ± 1.55 ^d^	<0.001
Oxyproline	25.3 ± 0.37 ^a^	23.0 ± 0.38 ^b^	20.7 ± 0.32 ^b^	18.4 ± 0.28 ^c^	16.1 ± 0.30 ^d^	13.8 ± 0.27 ^e^	<0.02
*Essential*	*6280 ± 113*	*5610 ± 100*	*6070 ± 109*	*5965 ± 107*	*5860 ± 105*	*5756 ± 103*	
Arginine	741 ± 14.9 ^a^	622 ± 6.43 ^b^	617 ± 8.08 ^b^	555 ± 8.42 ^c^	493 ± 9.02 ^d^	431 ± 6.22 ^e^	<0.001
Histidine	409 ± 5.03 ^b^	356 ± 4.26 ^b^	427 ± 6.67 ^b^	436 ± 6.87 ^b^	445 ± 7.82 ^a^	454 ± 6.57 ^a^	<0.02
Valine	754 ± 13.1	686 ± 7.15	738 ± 112	730 ± 9.76	722 ± 7.69	714 ± 5.38	<0.02
Isoleucine	588 ± 11.5	535 ± 8.08	592 ± 10.9	593 ± 7.15	595 ± 7.02	597 ± 6.89	>0.50
Leucine	1149 ± 22.8	1042 ± 18.1	1128 ± 18.9	1118 ± 15.9	1107 ± 16.1	1097 ± 13.5	>0.25
Lysine	969 ± 9.04	898 ± 16.4	994 ± 12.8	1006 ± 13.2	1018 ± 16.7	1030 ± 13.4	>0.25
Methionine	322 ± 5.05 ^a^	297 ± 2.65 ^b^	318 ± 4.71 ^a^	316 ± 4.58 ^a^	313 ± 5.15 ^a^	311 ± 4.89 ^a^	<0.01
Threonine	533 ± 6.07 ^a^	460 ± 5.44 ^b^	496 ± 8.46 ^a^	478 ± 6.20 ^b^	459 ± 8.25 ^b^	441 ± 6.89 ^b^	<0.02
Tryptophan	161 ± 2.25 ^a^	132 ± 1.68 ^b^	135 ± 2.32 ^b^	122 ± 1.77 ^b^	108 ± 1.70 ^b^	95.2 ± 1.23 ^c^	<0.001
Phenylalanine	654 ± 10.7 ^a^	583 ± 9.12 ^b^	627 ± 7.04 ^a^	613 ± 13.0 ^a^	599 ± 8.32 ^a^	586 ± 8.76 ^b^	<0.05
*Total Amino Acids*	*12360 ± 223*	*10605 ± 191*	*11154 ± 201*	*10551 ± 190*	*9948 ± 179*	*9345 ± 168*	

^a–d^ means within the same row with different uppercase letters differing significantly among different samples of liver pâtés (*p* < 0.05).

## Data Availability

The data presented in this study are available on request from the corresponding author.
